# Microdroplet surfaces interface organic molecule formation and the origins of life

**DOI:** 10.1016/j.xinn.2026.101278

**Published:** 2026-01-29

**Authors:** Shuai Chen, Nikolay Kornienko

**Affiliations:** 1 Institute of Inorganic Chemistry, University of Bonn, 53121 Bonn, Germany

## Main text

The primitive Earth, starting from the evolution of primitive chemistry, gradually developed into a habitable world. In prebiotic chemistry, a central question is how nature’s simplest molecules (e.g., carbon dioxide [CO_2_], water [H_2_O], and ammonia [NH_3_]) could form organonitrogen molecules, which serve as precursors to amino acids, nucleotides, and peptides. Urea, a representative organonitrogen molecule, typically requires extreme conditions in the industrial Bosch-Meiser process, including high pressure (125–250 bar) and high temperature (170°C–220°C).^1^ Those conditions are hardly simultaneously plausible on the primitive Earth.

**Figure 1:**
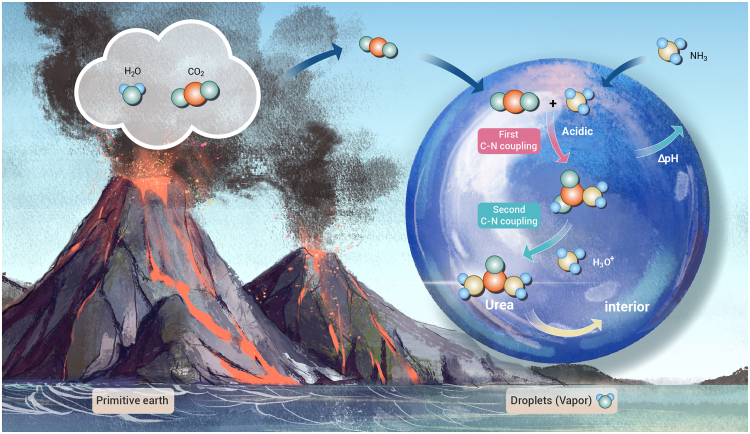
Droplets on the primitive Earth serving as prebiotic reactors for the spontaneous formation of urea under ambient conditions

In this remarkable recent study, Mercede et al. demonstrate that urea can spontaneously form within water droplets exposed to CO_2_ and NH_3_ gases under ambient temperature and pressure, without catalysts or external energy input.^2^ At the droplet interface, a microscopic flow reactor is established,^3^ where protons catalyze the coupling of CO_2_ and NH_3_ to generate urea. This nonequilibrium interfacial mechanism provides a plausible abiotic pathway for early organonitrogen chemistry. On the primitive Earth, geological processes such as volcanic eruptions would have released large amounts of H_2_O vapor and CO_2_, with the resulting droplets (H_2_O vapor) potentially enabling the spontaneous formation of urea from CO_2_ and ambient NH_3_ (Figure 1). Such findings reveal how simple, spontaneous physicochemical processes may have preceded enzymatic catalysis in the emergence of life.

To probe urea formation in a thousandfold-higher surface-to-volume ratio droplets, the authors employed *in situ* single-droplet Raman spectroscopy. In this setup, aerosol droplets were immobilized in a gaseous environment, and the focused Raman laser enabled time-resolved monitoring of interfacial species (e.g., urea and bicarbonate) over longer periods (many hours). This approach directly links the spectral evolution to interfacial chemistry within a microscopic reactor. Under a CO_2_ and NH_3_ atmosphere, a new Raman band appeared at 1,003 and 1,031 cm^−1^ after 30 min, corresponding to urea and bicarbonate species, respectively. Remarkably, the urea concentration reached 42 ± 10 mM, which is exceptionally high for an abiotic, catalyst-free system.^4^ Complementary *ex situ* experiments, including gas chromatography-mass spectrometry (GC-MS) and carbon-13 nuclear magnetic resonance (^13^C-NMR), further confirmed the successful synthesis of urea. In control experiments where CO_2_ was replaced by nitrogen (N_2_) gas, in the absence of dissolved ammonia or with bulk water, no urea was detected.

Quantum chemical calculations and reaction diffusion model revealed a two-step C–N coupling pathway responsible for spontaneous urea formation at the droplet surface. Compared with the bulk water, the droplet interface exhibits enrichment of H_3_O^+^ and strong pH gradients that create locally acidic conditions. These locally acidic conditions at the surface facilitate the first C–N coupling step to form neutral carbamic acid, followed by proton-catalyzed (H_3_O^+^) conversion to urea in the second C–N coupling step. The high surface-to-volume ratio of droplets establishes a dynamic flow reactor between the surface and interior. This dynamic flow reactor allows the continuous supply of reactants from the interior and the continuous removal of urea products from the surface. Looking forward, incorporating natural catalysts (e.g., minerals) into such droplets could further modulate interfacial pH, H_3_O^+^ concentration, and intermediate species, potentially enabling the formation of more complex organonitrogen compounds. Moreover, applying additional energy inputs (e.g., light, electricity, and thermal) could couple droplet interfacial chemistry to photo-, electro-, or thermochemistry pathways, thereby extending this mechanism toward more general prebiotic reaction networks.

For the origin of life, this work bridges atmospheric chemistry, interfacial chemistry, and primitive geological movement. On the primitive Earth, droplets generated from sea spray, volcanic eruptions, atmospheric condensation, and meteorite impacts were likely abundant. Each droplet could have acted as a flow reactor, driving the chemical evolution. Beyond CO_2_ and NH_3_, the primitive Earth’s atmosphere also contained methane (CH_4_), N_2_, hydrogen sulfide (H_2_S), and reduced phosphorus.^5^ Thus, beyond the spontaneous formation of urea, this mechanism could plausibly enable the spontaneous generation of other organonitrogen, organosulfur, and organophosphorus compounds. These compounds are the key components of amino acids, nucleotides, and other relevant biological building blocks. This discovery provides a plausible prebiotic route toward the spontaneous emergence of life-essential organic molecules. Therefore, the primitive Earth’s droplets may have served as both chemical incubators and ancestors of protocells.

Although the spontaneous formation of urea in droplets is both reasonable and remarkable, major challenges remain in bridging these laboratory observations with the complex environment of the primitive Earth. Understanding how such nonequilibrium interfacial chemistry could have linked with primitive chemistry remains an open question. Furthermore, extending this droplet-driven mechanism to other small molecules (e.g., H_2_S and reduced phosphorus) will be essential to determine whether similar pathways could give rise to broader families of prebiotic organonitrogen, organosulfur, and organophosphorus compounds. Ultimately, uncovering how these microscopic processes scaled up into the emergence of protocellular organization represents one of the great challenges in understanding the origin of life.

## Funding and acknowledgments

This work was supported by the 10.13039/501100008131University of Bonn. S.C. acknowledges funding from the Argelander Grant Starter-Kit (B) “Research Funds.”

## Declaration of interests

The authors declare no competing interests.
